# Data on the concentrations of etoposide, PSC833, BAPTA-AM, and cycloheximide that do not compromise the vitality of mature mouse oocytes, parthenogenetically activated and fertilized embryos

**DOI:** 10.1016/j.dib.2016.07.046

**Published:** 2016-07-30

**Authors:** Jacinta H. Martin, Elizabeth G. Bromfield, R. John Aitken, Tessa Lord, Brett Nixon

**Affiliations:** Priority Research Centre for Reproductive Science, Discipline of Biological Sciences and Hunter Medical Research Institute, University of Newcastle, Callaghan, NSW 2308, Australia

**Keywords:** Oocytes, Parthenote, Embryo, Vitality, Etoposide, PSC833, BAPTA-AM, Cyloheximide

## Abstract

These data document the vitality of mature mouse oocytes (Metaphase II (MII)) and early stage embryos (zygotes) following exposure to the genotoxic chemotherapeutic agent, etoposide, in combination with PSC833, a selective inhibitor of permeability glycoprotein. They also illustrate the vitality of parthenogenetically activated and fertilized embryos following incubation with the calcium chelator BAPTA-AM (1,2-Bis(2-aminophenoxy)ethane- N,N,N′,N′-tetraacetic acid tetrakis (acetoxymethyl ester)), cycloheximide (an antibiotic that is capable of inhibiting protein synthesis), and hydrogen peroxide (a potent reactive oxygen species). Finally, they present evidence that permeability glycoprotein is not represented in the proteome of mouse spermatozoa. Our interpretation and discussion of these data feature in the article “Identification of a key role for permeability glycoprotein in enhancing the cellular defense mechanisms of fertilized oocytes” (Martin et al., in press) [1].

**Specifications Table**

**Table:** 

Subject area	*Biology,*
More specific subject area	*Oocyte/zygote protective mechanisms against double strand break DNA damage*
Type of data	*Graph and figures*
How data was acquired	*Immunocytochemistry and immunoblotting*
Data format	*Analyzed*
Experimental factors	*Mouse oocytes and zygotes were treated with etoposide (100 µg/ml) for 15 min when appropriate. Those used in the examination of permeability glycoprotein (PGP) efflux activity were pretreated with either the PGP inhibitor PSC833 for 15 min (5 µM) or with BAPTA-AM (5 µM)), or cycloheximide (20 µg/ml) for 4 h during the allotted activation/fertilization period.*
Experimental features	*Mouse oocytes and spermatozoa were harvested and zygotes or parthenotes produced via IVF or strontium chloride chemical activation, respectively. Oocytes were treated with etoposide and PSC833 in combination (a selective inhibitor of PGP), BAPTA-AM or cycloheximide. The cytotoxicity of these drugs was evaluated by labeling of the cells with a standard vitality reagent for 15 min at 37 °C.*
Data source location	*N/A*
Data accessibility	*All relevant data are presented within this article*

**Value of the data**

• These data provide valuable insight into the maintenance of mature mouse oocyte and zygote vitality following genotoxic insult with etoposide (100 µg/ml); a chemotherapeutic agent that elicits a potent inhibition of topoisomerase II α action.
• Similarly, these data indicate that selective pharmacological inhibition of permeability glycoprotein (PGP) with PSC833 (5 µM), as well as incubation of oocytes in BAPTA-AM (5 µM) and cycloheximide (20 µg/ml) for periods of up to 4 h following insemination with spermatozoa or activation with strontium, does not adversely affect oocyte or embryo vitality.
• This information is of use to the scientific community as it establishes concentrations of various pharmacological reagents that can be utilized without compromising oocyte and embryo viability.
• Finally, these data provide evidence that mouse spermatozoa do not harbor PGP within their proteome, thus discounting the possibility of a paternal contribution to elevated levels of PGP found in the zygote.

## 1 Data

**Fig. 1:**
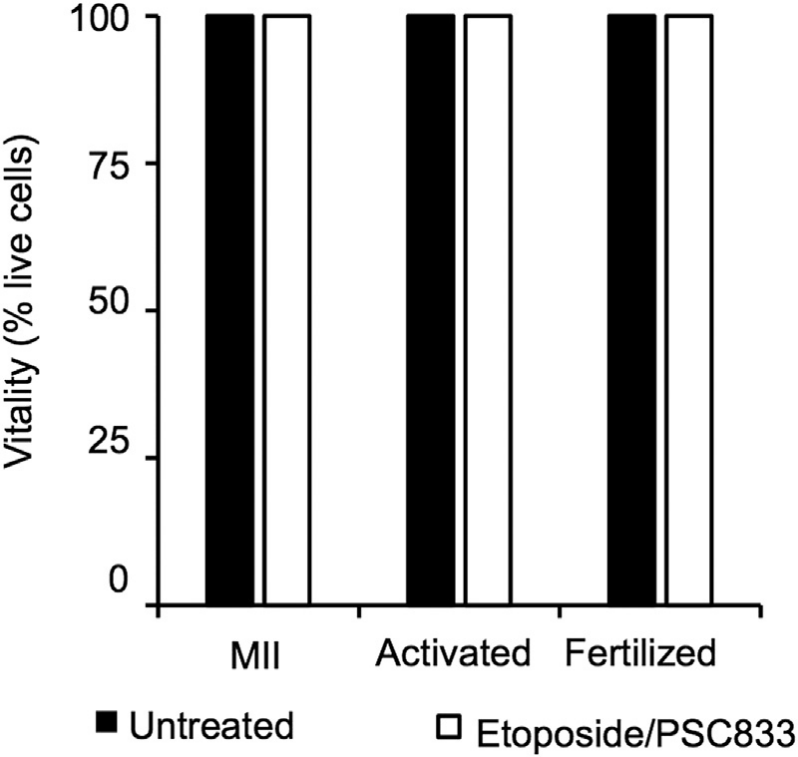
Co-incubation with etoposide and valspodar (PSC833), a selective inhibitor of PGP efflux activity did not see a loss in oocyte vitality. The cytotoxicity of etoposide and PSC833 were evaluated using as a standard far red detectable live/dead vitality reagent. Analysis confirmed that in no instance was there an associated decrease in oocyte vitality following treatment exposure *n*=3.

**Fig. 2:**
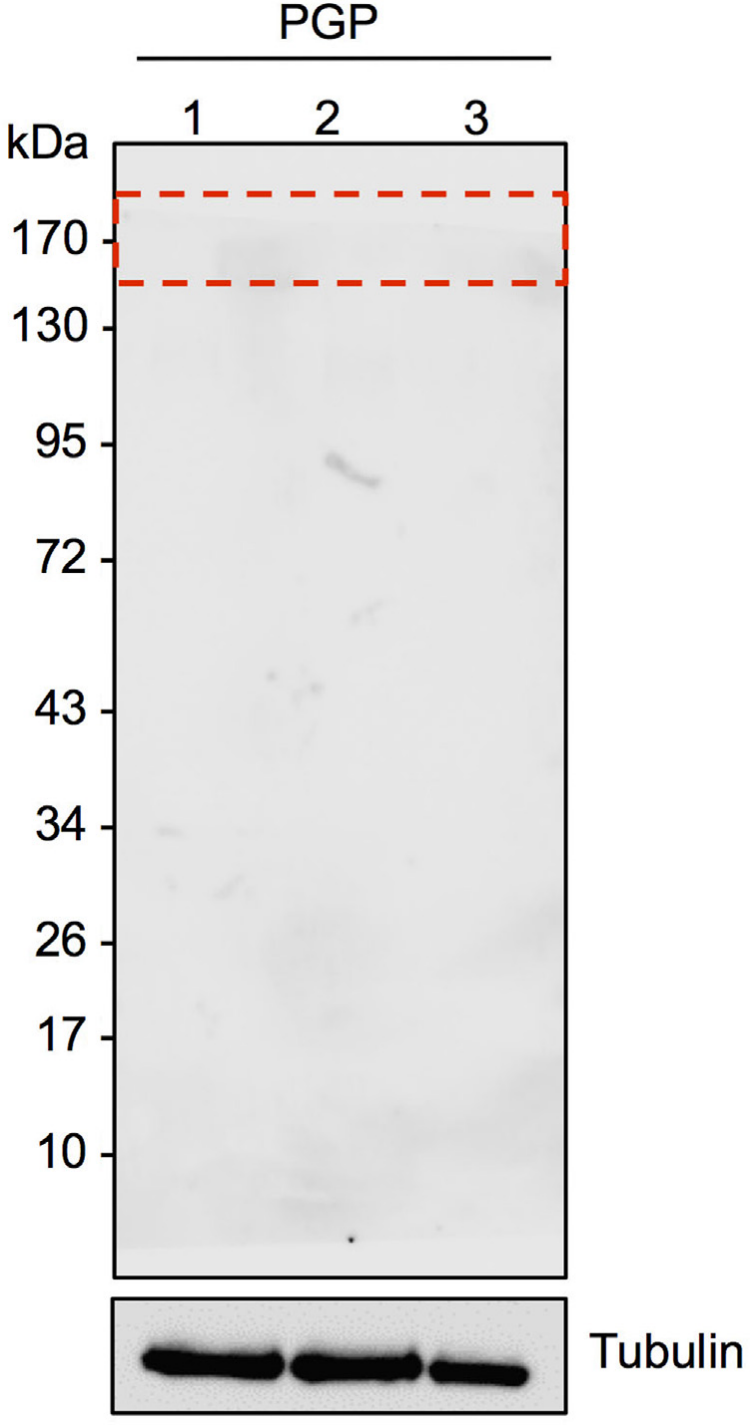
Permeability glycoprotein is absent from mature capacitated mouse spermatozoa. Immunoblotting techniques confirmed that permeability glycoprotein is absent from mature capacitated mouse spermatozoa; no bands were recorded over three replicates *n*=3.

**Fig. 3:**
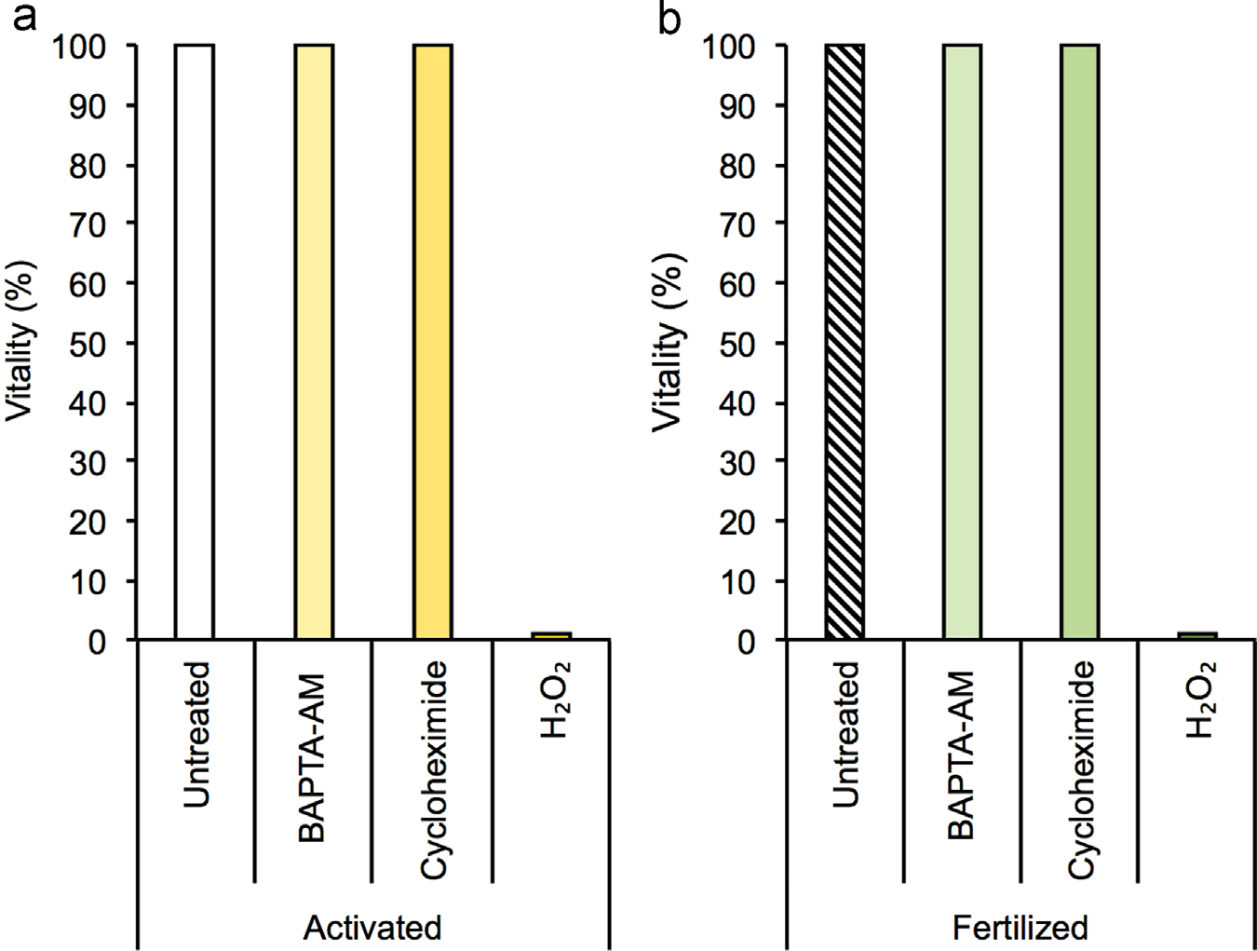
Loss of permeability glycoprotein expression is not an artifact of cell death (a,b). Immunofluorescence using a far red detectable live/dead vitality reagent indicated that oocyte and embryo vitality was conserved following treatment with cycloheximide, an antibiotic with the ability to block protein synthesis and the calcium chelator BAPTA (1,2-Bis(2-aminophenoxy)ethane-N,N,N′,N′-tetraacetic acid tetrakis (acetoxymethyl ester)), in the absence of PGP expression *n*=3.

The files included in this article comprise vitality profiles of mouse MII stage oocytes, chemically activated and fertilized zygotes following exposure to etoposide (100 µg/ml) in combination with PSC833 (5 µM) (Fig. 1), cycloheximide (20 µg/ml), BAPTA-AM (5 µM) or hydrogen peroxide (1 mM) (Fig. 3). This latter treatment was included as a positive control. Immunoblots of mouse sperm lysates with of anti-PGP antibodies are also included in this article (Fig. 2).

## 2 Experimental design, materials and methods

### 2.1 Reagents

Reagents were purchased from Sigma Aldrich (St Louis, MO, USA) unless otherwise stated. Anti-permeability glycoprotein (PGP; ab170904) antibody used for immunoblotting was procured from Abcam (Cambridge, England, UK).

### 2.2 Gamete retrieval

Gamete retrieval was achieved via superovulation of juvenile (3–5 week old) C57/BL6/CBA F1 female mice. Once primed, the mice were culled using CO_2_ asphyxiation and the oocytes recovered from the cumulus mass following incubation in hyaluronidase (300 µg/ml, 3–5 min) [2]. Spermatozoa were recovered from the cauda epididymides of mature (≥8 weeks) male C57/BL6/CBA F1 mice by retrograde perfusion via the vas deferens [3]. Sperm capacitation was achieved by incubation in modified Biggers, Whitten and Whittingham (BWW) medium supplemented with 1 mg/ml polyvinyl alcohol and 1 mg/ml methyl-beta cyclodextrin [2].

### 2.3 Strontium activation and *in vitro fertilization* (IVF)

Parthenogenic activation was stimulated by incubation of cumulus free oocytes in calcium free KSOM medium supplemented with 10 mM strontium chloride for 4 h. *In vitro* fertilization was carried out in human tubal fluid (HTF) medium containing 1 mM reduced glutathione (GSH). Recovered oocytes were co-incubated with 2×10^5^ capacitated spermatozoa for 4 h at 37 °C and successful fertilization (or activation in the case of parthenotes) was assessed by recording the extrusion of the second polar body and/or pronucleus formation [4].

### 2.4 Assessment of factors influencing permeability glycoprotein (PGP) expression

A number of drugs were used to investigate the factors responsible for the notable increase in PGP labeling following fertilization and activation [1]. The relative contribution of protein translation was analyzed by the inclusion of cycloheximide (20 µg/ml), an antibiotic with the ability to block protein synthesis. Alternatively, the role of activation-associated cytosolic calcium oscillations was assessed in the presence of the calcium chelator, BAPTA-AM (5 µM).

### 2.5 Etoposide treatment and cellular vitality

MII oocytes, parthenotes and pronuclear stage zygotes, were exposed to etoposide (100 µg/ml for 15 min at 37 °C), a topoisomerase II α inhibitor capable of eliciting DSB DNA damage. The cytotoxicity of etoposide, PSC833 [5], [6], BAPTA-AM and cycloheximide at the specific concentrations employed in this investigation were evaluated by labeling of the cells with a far red detectable live/dead vitality reagent (Thermo Fisher Scientific, Waltham, MA, USA).

### 2.6 Immunofluorescence, SDS PAGE and immunoblotting

Following retrieval and appropriate treatment, oocytes were washed in PBS/PVP and fixed by incubation in 3.7% (v/v) paraformaldehyde for 1 h at room temperature (RT). Oocytes were later mounted on Menzel Glӓser microscope slides (Thermo Fisher Scientific) in Mowiol containing 1,4-diazabicyclo[2.2.2]octane (DABCO) and fluorescence intensity assessed using an AXIO Imager.A1 fluorescence microscope (Carl Zeiss Micro Imaging GmbH, Jena, Thuringia, Germany). SDS PAGE and immunoblotting was conducted on solubilized (sodium dodecyl sulfate (SDS)) sperm protein extracts. A total of 10 µg of protein was resolved on pre-cast gels (4–12% NuPAGE BIS-Tris, Thermo Fisher Scientific) and transferred to a nitrocellulose membrane. Membranes were blocked in Tris-buffered saline (TBS) containing 0.1% Tween (TBST) with 3% BSA for 1 h) [7]. Blocked membranes were then sequentially incubated in anti-PGP primary antibody (1:500 in 3% BSA/TBST) overnight at 4 °C, HRP-conjugated secondary antibodies (1:1000 in 1% BSA/TBST) for 1 h at RT, stripped (western reprobe buffer in dH_2_O, Astral Scientific, Sydney, NSW, Australia) and reprobed with an anti-α-tubulin antibody (1:4000 in 1% BSA/TBST) to ensure equal protein loading.

### 2.7 Statistical analysis

Image processing was achieved using the public sector program, Image J (National Institute of Health, Bethesda, MD, USA). Statistical significance was determined using JMP software (version 10.0.0, SAS Institute, NC, USA). Each experiment was conducted on a minimum of three biological replicates and expressed as the mean±s.e.m. Differences with a value of *P*<0.05 were considered statistically significant.
